# The daily enactment of behavior change techniques among physically active university students with physical disabilities and chronic conditions

**DOI:** 10.1093/abm/kaaf065

**Published:** 2025-08-26

**Authors:** Gabrielle D Bedard, Olivia L Pastore, Jordan D Herbison, Jennifer R Tomasone, Tayah M Liska, Marley A R S Mullan, Alexandra Burns, Alan Jeans, Shane N Sweet

**Affiliations:** Department of Kinesiology and Physical Education, McGill University, Montreal, QC, H2W 1S4, Canada; Center for Interdisciplinary Research in Rehabilitation, Montreal, QC, H3S 1M9, Canada; Department of Kinesiology and Physical Education, McGill University, Montreal, QC, H2W 1S4, Canada; Center for Interdisciplinary Research in Rehabilitation, Montreal, QC, H3S 1M9, Canada; Department of Kinesiology, Vancouver Island University, Nanaimo, British Columbia, V9R 5S5, Canada; Kinesiology and Health Studies, Queen’s University, Kingston, Ontario, K7L 3N6, Canada; Department of Kinesiology and Physical Education, McGill University, Montreal, QC, H2W 1S4, Canada; Center for Interdisciplinary Research in Rehabilitation, Montreal, QC, H3S 1M9, Canada; Kinesiology and Health Studies, Queen’s University, Kingston, Ontario, K7L 3N6, Canada; Student Accessibility and Achievement Office, McGill University, Montreal, QC, H3A 0G3, Canada; Student Accessibility Service, Queen’s University, Kingston, Ontario, K7L 2N9, Canada; Department of Kinesiology and Physical Education, McGill University, Montreal, QC, H2W 1S4, Canada; Center for Interdisciplinary Research in Rehabilitation, Montreal, QC, H3S 1M9, Canada

**Keywords:** BCTs, behavior change, physical activity promotion, university students with disabilities, theory, experience sampling method (ESM)

## Abstract

**Background:**

While behavior change techniques (BCTs) support physical activity (PA) behavior change, further examination aimed at understanding their enactment is needed.

**Purpose:**

This study aimed to examine the daily enactment of BCTs and the factors that impact their daily usage.

**Methods:**

Using experience sampling methods, we sampled 53 university students (*M*_age_ = 22 years; SD_age_ = 3.93 years) from McGill (*n *= 40) and Queen’s (*n *= 13) University to examine BCT usage among university students with physical disabilities and chronic conditions. A daily online survey was delivered for 10 consecutive days where participants answered a checklist of 28 BCTs, a modified 6-item Capability, Opportunity, and Motivation (COM) questionnaire and questions on situational disruptions. Descriptive analysis summarized BCT usage, COM, and contextual factors. A generalized linear mixed model examined the relationship between demographic variables, COM, and contextual factors on the usage of BCTs.

**Results:**

On average, participants used 11 BCTs daily, while 22 distinct BCTs were used over the 10 days. The most frequently used BCTs were task crafting and goal integration. The least used BCTs included self-monitoring and obtaining information on how to perform PA. Women were more likely than men to enact certain BCTs, while students with physical disabilities were less likely than those with chronic conditions to enact others. Participants reported higher daily capability and opportunity to use BCTs compared to motivation. Situational disruptions such as personal commitments (eg, work) followed by flare-ups (eg, pain) were frequently reported. Motivation and opportunity predicted the usage of 15 to 24 BCTs, while capability predicted the usage of 2 BCTs.

**Conclusion:**

Our findings provide valuable context on BCT enactment, thereby improving the structure of future PA behavior change interventions.

## Introduction

According to Statistics Canada,^1^ 62% of Canadians with disabilities report pain-related limitations, 40% report flexibility disabilities, 30% report mobility issues, and 45% live with chronic conditions. Since living with disabilities can increase the risk of developing additional chronic conditions over time,^2^ there is growing recognition of the importance of promoting health behaviors like physical activity to mitigate such risks. Physical activity plays a key protective role in preventing secondary conditions, yet individuals with physical disabilities (eg, chronic pain, Ehlers-Danlos syndrome, amputation) and those with chronic conditions (eg, diabetes, asthma, endometriosis) are 18%-31% less likely to engage in physical activity than individuals without disabilities.^2^ The low enactment rate in physical activity is concerning, given the well-documented physical and psychological health outcomes for persons with disabilities,^2^ especially students.^3^ In fact, students with disabilities and chronic conditions represents one of the least physically active groups, with 72.2% Spanish students with physical disabilities not achieving the physical activity guidelines.^4^ Since university students with disabilities are a fast-growing proportion of the student population in Canada,^5^ we need to examine strategies that can support their physical activity participation.

Behavior change techniques (BCTs), which are intervention-based strategies designed to facilitate changes in health-related behaviors.^6^ In recent years, BCTs have been recognized for their ability to promote physical activity participation among individuals with disabilities.^7^ However, there is insufficient evidence to conclude the effectiveness of BCTs due to inconsistencies in the types and frequency of BCTs used in behavior change interventions.^7^^,^^8^ As such, a shift in focus from testing the impact of BCTs to investigating how participants understand and explicitly enact BCTs is needed.^9^ BCT enactment refers to how individuals apply the learned skills on their own in their everyday life to support behavior change. While some studies have begun this exploration,^9^^,^^10^ findings suggest that only 36% of participants consistently enacted all 16 BCTs that were taught in the intervention and 40.5% enacted all 8 BCTs to improve physical activity levels.^11^ However, many participants struggled to apply BCTs on their own beyond the intervention.^12–14^ The challenge of BCT enactment may be even more pronounced among individuals with physical disabilities and chronic condition, but little data on how BCTs are used among this population exist. Examining how students with disabilities and chronic conditions select and use self-enacted BCTs could elucidate the role of BCT in physical activity engagement for this group.^9^

Hankonen^9^ highlighted a need for more understanding and specific theoretical explanations about the enactment and frequency of BCTs. They argue that BCTs may have their own psychosocial predictors. The COM (Capability, Opportunity, and Motivation)-B model could be a valuable framework for understanding the antecedents of BCT enactment.^15^ The model proposes that to influence behavior (B), the individual must possess or develop the capability (C), opportunity (O) and motivation (M) to engage in the target behavior.^16^ In the context of BCT enactment, capability involves having the physical and psychological skills to enact BCTs. Opportunity refers to the physical and social factors that can impact the enactment of BCTs. Motivation encompasses reflective and automatic processes of BCT enactment.

Utilizing COM-B as the guided framework could offer insight into the relationship between these 3 components and the enactment of BCTs. For example, we need to examine whether individuals have the knowledge, skills, and abilities to engage in BCTs to understand if BCTs are too complex and cognitively challenging to be self-enacted.^14^ Moreover, using COM-B will allow us to examine social or physical opportunities predict BCT enactment, as it appears that social support may relate to BCT enactment.^13^ Additionally, if motivation is varied in BCT enactment,^13^ the COM-B model can examine how variations in motivation impact BCTs enactment. This theoretical approach will thereby enhance our understanding behind the mechanism of BCTs as they are enacted in real-life settings among university students with physical disabilities and chronic conditions.

### Purpose and research questions

This study examines the enactment of BCTs by physically active university students with physical disabilities and/or chronic conditions Therefore, the purpose of this study was to understand (1) how are BCTs enacted daily, and (2) what is the relationship between capability, opportunity and motivation, contextual factors, and the enactment of BCTs among physically active university students with physical disabilities and/or chronic conditions?

## Methods

### Design

The study used an intensive longitudinal design and experience sampling method (ESM).^17^ To be eligible to participate, students had to (1) have a physical disability and/or a chronic condition. Including both groups reflects the reality that many individuals experience overlapping or co-occurring health challenges, making it unrealistic to categorize them under a single label. This is particularly true for young adults with disabilities, who are 43% more likely than working-age adults to report multiple disabilities. By integrating both physical disabilities and chronic conditions, our approach acknowledges the multifaceted nature of disability as it is experienced in real time. Further (2) participants must be at least 18 years of age, (3) be from McGill or Queen’s University, (4) speak English or French, (5) have access to a smartphone device, and (6) engage in moderate-to-vigorous physical activity (MVPA) at least twice per week for a minimum of 30 minutes in the last 3 months. Given the limited evidence on how BCTs are enacted to support physical activity, we recruited physically active students who are most likely already enacting them. Given the discrepancy in physical activity guidelines for people with disabilities,^18^ the final inclusion criteria followed an adapted version of the physical activity guidelines^19^ to better align with the targeted population of our study. The chosen threshold ensure that the level of physical activity is realistic and reflective to what is achievable for individuals with disabilities.^18^ Exclusion criteria included students who identified having a mental health disorder, neurodevelopmental or sensory disabilities. All data were collected from June 2023 to March 2024.

The targeted sample size minimum of 40 participants was based on previous ESM studies where studies with more than 15 participants enhanced the likelihood of finding significant results.^20^ However, considering potential attrition rates, we expected a final sample of 40 participants. Attrition rates were calculated based on previous physical activity studies of students participating in EMA studies. One study reported that 11% did not complete the study,^21^ while another reported an attrition rate of 32%.^22^ By averaging these rates, we expected an attrition rate of approximately 21%, representing 10 participants who were expected to drop out.

Fifty-three university students (*M*_age_ = 22 years, SD_age_ = 3.93 years) who met the inclusion criteria were recruited to participate in the ESM study. Participants who reported a physical disability (accounting for 23% of our sample) experienced limitations in function, coordination, or strength in one or more limbs due to injury, surgery, or hereditary factors. Those reporting a chronic condition (accounting for 85% of our sample) had a long-term health condition requiring ongoing care. Given that 79% of Canadians report multiple disabilities, with young adults more likely to experience 2 or more types of disabilities,^1^ and the rise of secondary health disorders,^2^ both categories were acknowledged in this study.

### Procedures

Ethics approval was obtained for this study from McGill (R2EB # 22-06-095) and Queen’s (TRAQ # 6038156) University Research Ethics Boards, and informed consent was obtained from all individuals who participated in the study. A convenience sampling method was utilized to reach potential students. The Students Accessibility and Achievement Services from McGill University distributed hard copies of the study poster within their service center, shared the recruitment poster on their social media platform, and emailed the study information to their respective email list. The research team also contacted respective professors from McGill and Queen’s University, requesting their collaboration in sharing the study recruitment with students on their online course platform.

Participants interested in the study accessed a screening questionnaire via a link or QR code. The link was available through recruitment posters social media, email, or online course platform. The screening questionnaire collected the student’s name, university emails, and eligibility information. Ineligible participants were notified immediately. The research team examined each participant who was deemed eligible based on the screening form to ensure eligibility.

Once participants were deemed eligible, the research team emailed each participant their unique ID code, an invitation to a prestudy meeting, and a link to access the consent form and baseline questionnaire. Once consent was provided and the baseline questionnaire was completed, participants were enrolled in a 10-day data collection period. Each day, the participants received a randomized pin notifications on their smartphones between 4:00 and 8:00 pm, prompting them to complete the daily questionnaires, which then remained open until 2:00 am. The daily prompts directed participants to self-report their physical activity, BCTs, COM-B, and information regarding situational disruptions. Completing the daily questionnaires took approximately 5-8 minutes to complete.

#### Prestudy meeting

During the prestudy meeting, the researcher verified the consent form and baseline questionnaire were completed and answered questions about the study procedures.

During the prestudy meeting, the research team introduced Pathverse^23^ and ensured that participants were familiar with the app. Pathverse is a mobile health behavior change app designed to help researchers by tracking recruitment, participant progress, and engagement. Developed with an ESM framework, it allowed the research team to customize and schedule daily randomized pin notifications, set survey closing times, and build the daily survey cards. In the study, Pathverse was used to deliver randomized notifications over a 10-day period, prompting participants to complete daily surveys on their physical activity, BCTs COM-B factors, and situational disruptions. A screen recording on how to navigate the app and its features was presented during the prestudy meeting to familiarize participants with the mobile application. Once participants were comfortable navigating the app, they selected a start date for the study.

#### ESM daily survey protocol

The daily questionnaires (ie, BCT, COM-B, Physical Activity, and Situational information) were delivered through the Pathverse mobile application, which sent daily randomized single-pin notifications on their smartphones between 4:00 pm and 8:00 pm to minimize response bias. Participants had until 2:00 am to complete the daily survey. Participants were compensated up to $75 for participating in the study.

### Measures

#### Demographic and baseline physical activity information

Participants provided details on their demographic background in the baseline questionnaire only. Questions included (1) month and year of birth, (2) gender, (3) ethnicity, (4) university institution, (5) school faculty, (6) type of housing, (7) time of commute to school, (8) average work hours, (9) type of physical disabilities and/or chronic condition, (10) reliability to move around the community, and (11) primary mode of mobility. The self-reported leisure time physical activity (LTPA) questionnaire was used to describe the sample’s physical activity levels at baseline.^24^ For each type of physical activity (ie, aerobic and strength), participants were asked to estimate duration for each intensity (ie, mild, moderate, and vigorous) in the last 7 days. Physical activity levels were then reported as the mean number of minutes in the last 7 days.

#### BCT questionnaire

The study used a modified version of a BCT questionnaire.^12^ Both the baseline questionnaire and daily surveys presented participants with descriptions of 28 selected BCTs. The 28 BCTs were identified through a systematic selection process.

##### BCT systematic selection process

Given the large number of BCTs in the literature and the high participation burden of measuring all BCTs, the research team narrowed the BCT list for our targeted population through a 4-step approach (Table S3).

First, the research team conducted a literature search of meta-analyses/reviews (*n* = 26) of physical activity intervention and identified the 8 BCTs that were consistently used across physical activity interventions. Second, each of the 5 team members consulted the self-enactable BCT compendium list^25^ and selected 10 self-enactable BCTs from the list as most relevant to our target sample. Based on the team discussion and debriefing, 5 additional BCTs were selected making our total to 13 BCTs. Third, we examined the 13 selected BCTs through the lends of the Behavior Change Wheel^16^ to ensure that we would select BCTs that are linked to theoretical constructs of behavior change. Specifically, we aligned each BCT to the COM-B model using the larger subset of theoretical variables of Theoretical Domain Framework (ie, framework with 14 domains designed to identify factors influencing behavior change).^26^ Given the 13 selected BCTs only covered a portion of the theoretical domains, we consulted Richardson et al (ie, that aligns BCTs with Theoretical Domain Framework and COM-B)^27^^,^^28^ to identify 15 additional BCTs to ensure all theoretical domains of the TDF were represented. Finally, we transformed the definitions of the 28 BCTs into lay terms.

To ensure the vocabulary was not difficult to comprehend, we conducted a university-scale readability assessment. Afterwards, 1 PhD student, 1 master’s student, and 1 undergraduate student who were unfamiliar with these techniques were asked to match the new definitions to the appropriate BCTs. After revising some definitions, the same PhD ran a second trial to ensure that the revised BCT definitions were clear and accurate. We then carried out 2 pilot studies with university students with disabilities to gather their feedback on their understanding of the BCTs daily questionnaires. The 2 pilot studies helped to ensure that the lay description of the BCTs was not difficult and accessible for our target population.

##### BCT measures

Participants were presented the name of the BCT and a lay description during the daily surveys. Examples include: (1) Task crafting, *“*I chose a physical activity that matches my skills and ability (eg, weights that are not too heavy or too light)”; (2) Goal integration, “I did my physical activity at the same time as another personal interest (eg, I walked while listening to music)”; (3) Graded tasks, “I started with a simple physical activity and gradually made it harder (eg, I gradually increased the intensity of my physical activity)”; (4) Self-monitoring, “I kept track of my physical activity (eg, I wrote down my physical activities in a diary)”; and (5) Behavioral self-praise, “I verbally rewarded myself after I did my physical activity (eg, I said to myself ‘Great job!’).” For the baseline questionnaire, participants’ enactment of 28 BCTs in the last 2 weeks was assessed using a 5-point Likert scale ranging from 0 (not even once) to 5 (daily). In the daily survey, participants responded yes or no to whether they used each of the 28 techniques in the last 24 hours.

#### COM-B questionnaire

The study used a modified COM-B questionnaire version from a generic 6-item self-evaluation COM questionnaire^29^^,^^30^ to measure the student’s capability, opportunity, and motivation to use BCTs in physical activity both at baseline and throughout the daily surveys. The COM-B questionnaire measured the subcomponents of COM-B, such as physical and psychological capability, physical and social opportunity, and automatic and reflective motivation. Each subcomponent was assessed on a 10-point Likert scale ranging from 0 (strongly disagree), to 10 (strongly agree). The baseline questionnaire aimed to measure the participants’ general perception of their capability, opportunity, and motivation of using BCTs in physical activity, while the daily survey measured their COM-B over subsequent 24-hour periods. An example of a capability statement for the daily surveys includes, “I was physically able to use behavior change strategies in my physical activity today.” An example of opportunity in the daily surveys is, “I had the social opportunity to use behavior change strategies in my physical activity today.” An example of motivation in the daily surveys is, “I was motivated to use behavior change strategies in my physical activity today.” The overall score for capability, opportunity, and motivation was created by calculating the mean of the 2 items for each respective subcomponent.

#### Situational disruption information

The daily survey asked participants 5 questions about contextual factors that might have disrupted their engagement in physical activity BCTs. Participants responded to either yes or no to factors such as exams or course assignments, illness, weather, flare-ups, other personal responsibilities, or commitments.^4^^,^^27^^,^^28^

### Multilevel analytic strategy

Given our experience with and user-friendly interface, we used SPSS 29.0.0.0^29^^,^^30^ to conduct our data cleaning process and analyze descriptive statistics for the sociodemographic variables, LTPA, BCTs, COM-B, and situational disruption information. Once the dataset was cleaned and descriptive analyses conducted in SPSS, we used ChatGPT 4o^31^ to examine the frequency of BCT combinations to provide a more detailed descriptive understanding of BCT enactment. To answer our second research question, we imported the cleaned data in R 4.3.1^32^ to run the multilevel model using the “Lme4” package and the “glmer” and “binomial” function.^32^ The repeated measurement of participants’ perceptions resulted in a 2-level nested, time-ordered, and multivariate data structure. A total of 84 separate generalized linear mixed models (GLMMs) were run to model the within-person relationships between each of the 28 BCTs (as outcome variable) and 3 sets of predictors: sociodemographic variables (ie, 28 models), COM variables (ie, 28 models), and situational disruptions (ie, 28 models). We used a random intercept model to allow the intercept to vary across individuals. Using the *“*exp(fixef)” function,^33^ coefficients expressed in log-odds were transformed to odd ratios for predictor interpretation in the likelihood of using BCTs. Continuous predictors (COM variables) showed odds changes per unit increase, while binary predictors (contextual factors) indicated the change in the odds of using BCTs when the predictor changes from 0 to 1.^34^

## Results

Two hundred and twenty-seven students completed the screening questionnaire to participate in our study. One hundred and seventy-four students were ineligible, and 1 student who expressed interest was unable to participate due to time constraints. A total of 53 participants enrolled in the study. All participants completed at least 3 days, 86.8% provided at least 7 days of data, and 30.2% completed all 10 days. The 53 participants (*M*_age_ = 22 years, SD_age_ = 3.93 years) were current students from McGill (75%) and Queen’s (25%) University. Most self-identified as a woman (68%), and reported having chronic conditions (85%), pain-related disabilities (23%), and/or mobility/flexibility disabilities (19%) (Table 1).

**Table 1.** Sociodemographic characteristics of participants at baseline.^a^

	Baseline characteristic	*n* (%)	*M* (SD)
Gender	Man	14 (26)	
	Non-binary	<5 (9)	
	Transgender/intersex	<5 (9)	
	Woman	36 (68)	
Ethnicity	Arab	<5 (9)	
	Black	<5 (9)	
	Chinese	7 (13)	
	First Nations	<5 (9)	
	Latin American	5 (9)	
	South Asian	7 (13)	
	West Asian	<5 (9)	
	White	30 (57)	
Disabilities	Chronic conditions	45 (85)	
	Mobility/flexibility	10 (19)	
	Pain-related	12 (23)	
Transportation aid	No assistance	47 (89)	
	Personal assistance	6 (11)	
University	McGill University	40 (75)	
	McGill University	13 (25)	
University level	Professional/graduate	11 (21)	
	Undergraduate	42 (79)	
Faculty	Arts and Humanities	18 (34)	
	STEM	14 (26)	
	Education and Health/Social Sciences	21 (40)	
Commute	0-30 minutes	39 (73)	
	31-60 minutes	8 (15)	
	Over 60 minutes	5 (9)	
	N/A	<5 (9)	
Work hours	Less than 15 hours per week	42 (81)	3.96 (4.91)
	Less than 35 hours per week	5 (9)	22 (7.49)
	More than 35 hours per week	6 (11)	44 (9.17)
LTPA total (minutes/day)			49.11 (10.55)
MVPA total (minutes/day)			41.64 (6.89)

a *n *= 53. *M*_age_ = 22, SD_age_ = 3.93 Some percentages exceed 100% since participants were allowed to select more than 1 option. LTPA and MVPA represent the daily average over a 10-day period.

Abbreviations: LTPA, leisure time physical activity; MVPA, moderate-to-vigorous physical activity.

Outliers for all variables were initially identified and handled in SPSS using box plots. A Winsorization technique was applied for the LTPA variable to adjust the outliers on SPSS. Outliers below the 5th percentile and above the 95th percentile were replaced with values just within the percentile limit to reduce the impact of extreme values.^29^^,^^30^ A multicollinearity (ie, variance inflation factor) test in R was conducted for the COM variables.^32^ We did not impute missing data in the GLMMs considering its resilience in handling missing observations in the outcomes.^35^ However, since LTPA was not a variable within the GLMM analysis, missing completely at random test, and mean imputation were performed for missing data (ie, 5 data points).

### Research question 1: descriptive statistics of BCTs

On average, physically active participants reported using 11 BCTs per day (SD = 5.53, R = 1-27). Across the 10 days, participants, on average, used 22 of the 28 BCTs (SD = 5.14, R = 6-28). The most frequently used BCTs were task crafting with *n = *52 participants using it for, on average, 6 days (R_days_ = 0-10) over the 10 days, followed by goal integration (*n *= 51, *M*_days_ = 5, R_days_ = 0-10), and finding meaning in physical activity *(n *= 50, *M*_days_ = 5, R_days_ = 0-10). The least frequently used BCTs included obtaining information on how to perform physical activity (*n *= 23, M_days_ = 2, R_days_ = 0-10), pros and cons (*n *= 26, *M*_days_ = 1, R_days_ = 0 -7), and self-monitoring (*n *= 33, *M*_days_ = 3, R_days_ = 0-10) (Table 2).

**Table 2.** Physical activity BCT frequency and percentage among participants over 10 days.^a^

BCTs	Count	Participants using BCT (%)	Average BCT frequency of participants
Task crafting	301	52 (98)	6 (R = 1-10)
Goal integration	284	51 (96)	5 (R = 0-10)
Finding meaning in physical activity	254	50 (94)	5 (R = 0-10)
Positive reframing	251	49 (92)	5 (R = 0-10)
Restructuring the social environment	203	49 (92)	4 (R = 0-9)
Focus on enjoyment	276	48 (91)	5 (R = 0-10)
Normalizing difficulty	210	48 (91)	4 (R = 0-10)
Behavioral goals	228	47 (89)	4 (R = 0-9)
Graded task	192	46 (87)	4 (R = 0-9)
Self-kindness	194	45 (85)	4 (R = 0-9)
Outcome goals	188	44 (83)	3 (R = 0-9)
Restructuring the physical environment	196	42 (79)	4 (R = 0-9)
Self-talk	184	42 (79)	3 (R = 0-10)
Reflection on the need to perform physical activity	179	42 (79)	3 (R = 0-9)
Emotional support	167	42 (79)	3 (R = 0-9)
Problem solving	164	42 (79)	3 (R = 0-9)
Focus on past success	160	42 (79)	3 (R = 0-10)
Behavioral self-reward	160	42 (79)	3 (R = 0-9)
Prompt and cues	143	41 (77)	3 (R = 0-9)
Observe demonstration of physical activity	141	40 (75)	3 (R = 0-8)
Action planning	134	39 (74)	2 (R = 0-10)
Behavioral self-praise	145	36 (69)	3 (R = 0-10)
Obtaining information about antecedents	116	36 (68)	2 (R = 0-9)
Obtaining information about health consequences	106	35 (66)	2 (R = 0-8)
Practical social support	102	34 (64)	2 (R = 0-8)
Self-monitoring	144	33 (62)	3 (R = 0-10)
Pros and cons	73	26 (49)	1 (R = 0-7)
Obtaining information on how to perform physical activity	103	23 (43)	2 (R = 0-10)

Abbreviations: BCTs, behavior change techniques; R, range.

a *n* = 530. Missing values = 79 (15%).

**Figure 1.**
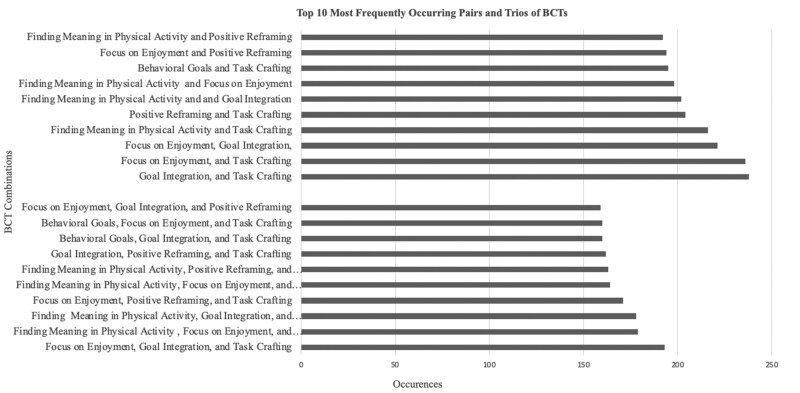
Top 10 most frequently occurring pairs and trios of enacted BCTs across 10 days. BCTs, behavior change techniques. The chart demonstrates the most common pairs of BCTs based on their frequency of occurrences across the 10 days. The total occurrences counted is *n* = 534.

The most common pairs of BCTs were (1) goal integration and task crafting (238 occurrences), (2) focus on enjoyment and task crafting (236 occurrences), and (3) focus on enjoyment and goal integration (221 occurrences) (Figure 1). The most frequent trios of BCTs used by participants day were (1) focused on enjoyment, goal integration, and task crafting (193 occurrences), (2) finding meaning in physical activity, focus on enjoyment, and task crafting (179 occurrences), and (3) find meaning in physical activity, goal integration, and task crafting (178 occurrences) (Figure 1).

### Research question 2: GLMMs of behavior change techniques enactment

The intraclass correlation coefficient was .271 indicating that 72.9% of the variability in BCT use was within-person, while 27.1% was between-person variability. Therefore, multilevel modeling was warranted as most of the variability in BCTs usage is within individuals.

Pertaining to demographic variables, gender, ethnicity/race, and chronic condition/disability were found to predict some BCT enactment (Table S1). Specifically, participants who self-identified as women were more likely to report using 13 BCTs (eg, obtaining information about health consequences, prompt and cues) compared to participants who self-identified as men (OR_gender_ range = 3.97-15.84, *P* < .05). Participants who identified as racialized individuals were more likely to use 4 BCTs (eg, obtaining information about health consequences, behavioral self-praise) compared to individuals who self-identified as white (OR_race_ range = 0.11-0.25, *P* < .05). Participants who reported having a physical disability were less likely to use 2 BCTs, restructuring of social environment (OR = 0.35, *P* < .05) and prompt and cues (OR = 0.30, *P* < .05) compared to participants who reported having a chronic condition.

Regarding situational disruptions that may impact their BCT engagement, participants reported, on average, at least 1 contextual factor per day (*M* (SD) = 1 (0.53), R = 0-4) and a total of 4 distinct factors (SD = 3.62, R_days_ = 1-5) across 10 days. The most frequently reported contextual factor was encountering commitments with 34 participants reporting it, on average, 3 of 10 days (R_days_ = 0-8), followed by flare-ups at (*n *= 35, *M*_days_ = 2, R_days_ = 0-10). Other reported contextual factors were exams (*n *= 33, *M*_days_ = 2.39), weather (*n *= 32, *M *= 2.12), and illness (*n *= 24, *M*_days_ = 2.24). Participants who reported in having commitments were less likely to use 15 BCTs (eg, behavioral goals, task crafting, and self-talk) compared to those who did not report having commitments (OR range = 0.34-0.56). Participants who reported having flare-ups were less likely to use 9 BCTs (eg, task crafting, problem solving, and prompt and cues) compared to those who did not report having flare-ups (OR range = 0.36-0.58). Participants who reported having an illness were more likely to use task crafting (OR = 1.95), and less likely to use finding meaning in their physical activity (OR = 0.32). Both exams and weather did not show any significant relationship with any of the 28 BCTs (Table S2).

As it pertains to daily capability, opportunity, and motivation in using BCTs for their physical activity, participants appeared to report higher mean levels of daily capability (*M*_days_= 6.42, SD = 2.23) and opportunity (*M*_days_ = 6.30, SD = 2.34) to use BCTs than motivation (*M*_days_ = 5.56, SD = 2.35). After controlling for significant demographic and contextual factors, all BCTs, except for pros and cons, were predicted by motivation, opportunity, or capability. Motivation and opportunity predicted the same 13 BCTs while no BCTs were predicted by all 3 constructs. Motivation was a significant predictor of 25 BCTs and was positively associated with a 16.6%-62.2% increase odds of using those BCTs. There were 5 BCTs that had at least a 40% increase chance of being used due to motivation: self-talk (OR = 1.62, SE = 0.10), graded tasks (OR = 1.57, SE = 0.09), obtaining information about health consequences (OR = 1.58, SE = 0.10), reflection on the need to perform physical activity (OR = 1.47, SE = 0.09), finding meaning in physical activity (OR = 1.44, SE = 0.09), and self-monitoring (OR = 1.43, SE = 0.11). Opportunity was also a significant predictor of 15 BCTs, with 20%-54.65% increased odds in using those BCT. There were 4 BCTs that had at least a 40% increase chance of being used due to opportunity such as emotional support (OR = 1.55, SE = 0.10), restructuring the social environment (OR = 1.47, SE = 0.09), practical social support (OR = 1.44, SE = 0.11), and self-monitoring (OR = 1.41, SE = 0.12). Capability was significantly and positively associated 2 BCTs: behavioral goals and problem solving. Neither of the 2 BCTs had at least 40% increase chance of being used due to capability (Table 3).

**Table 3.** Odds ratios and percentage changes for COM (Capability, Opportunity, and Motivation) predictors in behavior change techniques from generalized linear mixed model.^a^

Model	Predictors	Estimate	SE	*Z* value	Odd ratios (%)
Self-monitoring	(Intercept)	−5.71	1.30	−4.38^b^	0.00 (99.67)
	Capability	−0.17	0.13	−1.33	0.85 (15.44)
	Opportunity	0.35	0.12	2.90^c^	1.41 (41.50)
	Motivation	0.35	0.11	3.12^c^	1.43 (42.54)
	Commitment	−0.29	0.41	−0.70	0.75 (25.00)
	Gender	3.29	1.16	2.83^c^	26.95 (100)
	Ethnicity	−2.72	0.99	−2.75^c^	0.07 (93.44)
Obtain information about health consequences	(Intercept)	−4.94	0.93	−5.29^b^	0.01 (99.30)
	Capability	−0.18	0.11	−1.63	0.84 (16.20)
	Opportunity	0.10	0.10	1.01	1.11 (10.80)
	Motivation	0.45	0.10	4.45^b^	1.58 (57.50)
	Gender	2.57	0.70	3.67^b^	13.00 (100)
	Ethnicity	−1.84	0.57	−3.21^c^	0.16 (84.00)
Antecedents	(Intercept)	−4.39	0.83	−5.29^b^	0.01 (98.80)
	Capability	−0.14	0.11	−1.31	0.87 (13.20)
	Opportunity	0.28	0.10	2.73^c^	1.32 (32.40)
	Motivation	0.27	0.09	2.87^c^	1.31 (30.70)
	Gender	0.64	0.62	1.04	1.90 (90.10)
Task crafting	(Intercept)	−3.09	0.61	−5.03^b^	0.05 (95.50)
	Capability	0.18	0.09	1.92	1.20 (19.90)
	Opportunity	0.22	0.08	2.60^c^	1.25 (24.50)
	Motivation	0.30	0.08	3.52^b^	1.35 (34.60)
	Illness	0.67	0.45	1.50	1.95 (95.10)
	Flare-ups	−0.47	0.35	−1.32	0.63 (37.50)
Practical support	(Intercept)	−6.81	1.09	−6.27^b^	0.00 (100)
	Capability	0.19	0.12	1.56	1.23 (23.00)
	Opportunity	0.31	0.11	2.89^c^	1.36 (36.00)
	Motivation	0.11	0.10	1.12	1.17 (17.00)
	Gender	1.42	0.66	2.15^d^	4.14 (314.00)
Obtain information on how to perform physical activity	(Intercept)	−5.06	0.94	−5.38^b^	0.01 (99.40)
	Capability	0.07	0.12	0.59	1.07 (7.30)
	Opportunity	0.23	0.11	2.12^d^	1.26 (25.90)
	Motivation	0.24	0.11	2.26^d^	1.27 (27.30)
	Commitment	−0.69	0.38	−1.83	0.50 (50.10)
Restructuring the social environment	(Intercept)	−3.96	0.75	−5.26^b^	0.02 (98.09)
	Capability	−0.08	0.09	−0.85	0.92 (7.54)
	Opportunity	0.39	0.09	4.28^b^	1.47 (47.15)
	Motivation	0.23	0.08	2.75^c^	1.26 (25.62)
	Flare-ups	0.09	0.37	0.24	1.09 (9.36)
	Gender	1.63	0.57	2.84^c^	5.11 (100)
	Disability	−1.57	0.51	−3.07^c^	0.21 (79.25)
Restructuring the physical environment	(Intercept)	−5.04	0.93	−5.41^b^	0.01 (99.40)
	Capability	0.03	0.10	0.26	1.03 (2.60)
	Opportunity	0.29	0.09	3.16^c^	1.34 (33.50)
	Motivation	0.30	0.09	3.32^b^	1.35 (34.70)
	Commitment	−0.23	0.33	−0.69	0.80 (20.20)
	Gender	1.54	0.66	2.32^d^	4.64 (100)
Prompt and cues	(Intercept)	−4.30	0.87	−4.92^b^	0.01 (98.60)
	Capability	0.04	0.10	0.35	1.04 (3.60)
	Opportunity	0.07	0.09	0.75	1.07 (7.20)
	Motivation	0.32	0.09	3.39^b^	1.38 (37.80)
	Flare-ups	−0.36	0.40	−0.89	0.70 (30.00)
	Commitment	−0.75	0.33	−2.31	0.47 (53.00)
	Gender	2.40	0.63	3.78^b^	11.04 (100)
	Disability	−1.52	0.54	−2.80^c^	0.22 (78.20)
Behavioral goals	(Intercept)	−3.19	0.59	−5.45^b^	0.04 (95.90)
	Capability	0.25	0.09	2.74^c^	1.28 (28.30)
	Opportunity	0.09	0.08	1.14	1.09 (9.30)
	Motivation	0.23	0.08	2.89^c^	1.26 (25.70)
	Commitment	−0.69	0.30	−2.34^d^	0.50 (50.00)
Outcome goals	(Intercept)	−3.58311	0.57	−6.26^b^	0.03 (97.22)
	Capability	−0.02813	0.09	−0.30	0.97 (2.77)
	Opportunity	0.25269	0.09	2.94^c^	1.29 (28.75)
	Motivation	0.29383	0.08	3.54^b^	1.34 (34.16)
Goal integration	(Intercept)	−2.23	0.54	−4.13^b^	0.11 (89.20)
	Capability	0.12	0.09	1.39	1.13 (12.90)
	Opportunity	0.15	0.08	1.93	1.17 (16.80)
	Motivation	0.23	0.08	2.84^c^	1.26 (25.50)
	Commitment	−0.06	0.29	−0.22	0.94 (6.30)
Positive reframing	(Intercept)	−2.23	0.72	−3.09^c^	0.11 (89.20)
	Capability	0.17	0.09	1.89	1.19 (18.90)
	Opportunity	−0.05	0.08	−0.58	0.95 (4.60)
	Motivation	0.18	0.08	2.23^d^	1.20 (20.30)
	Flare-ups	−0.10	0.37	−0.26	0.91 (9.20)
	Gender	1.07	0.59	1.80	2.92 (100)
Reflect on the need to perform physical activity	(Intercept)	−3.34	0.66	−5.05^b^	0.04 (96.50)
	Capability	−0.09	0.10	−0.84	0.92 (8.30)
	Opportunity	0.18	0.09	1.98^d^	1.20 (20.20)
	Motivation	0.38	0.09	4.06^b^	1.47 (46.90)
	Commitment	−0.16	0.32	−0.49	0.86 (14.50)
Pros and cons	(Intercept)	−3.929	0.83	−4.76^b^	0.02 (98.03)
	Capability	0.065	0.12	0.53	1.07 (6.71)
	Opportunity	0.134	0.12	1.10	1.14 (14.39)
	Motivation	−0.009	0.11	−0.08	0.99 (0.88)
Self-praise (behavior)	(Intercept)	−4.72	1.03	−4.59^b^	0.01 (99.10)
	Capability	0.20	0.11	1.74	1.22 (21.60)
	Opportunity	0.04	0.10	0.42	1.04 (4.20)
	Motivation	0.25	0.10	2.53^d^	1.29 (28.90)
	Commitment	−0.35	0.35	−1.00	0.71 (29.50)
	Gender	2.27	0.90	2.51^d^	9.68 (100)
	Ethnicity	−2.23	0.79	−2.82^c^	0.11 (89.30)
Normalizing difficulty	(Intercept)	−1.98	0.52	−3.78^b^	0.14 (86.20)
	Capability	−0.01	0.09	−0.11	0.99 (1)
	Opportunity	0.06	0.08	0.81	1.06 (6.40)
	Motivation	0.28	0.08	3.58^b^	1.32 (32.40)
	Commitment	−0.17	0.28	−0.62	0.84 (15.80)
Focus on enjoyment	(Intercept)	−3.54	0.69	−5.11^b^	0.03 (97.10)
	Capability	0.15	0.10	1.54	1.16 (15.70)
	Opportunity	0.30	0.09	3.41^b^	1.35 (35.40)
	Motivation	0.26	0.09	2.91^c^	1.30 (29.50)
	Commitment	−0.05	0.32	−0.15	0.95 (4.80)
	Flare-ups	−0.09	0.37	−0.25	0.91 (8.70)
Self-kindness	(Intercept)	−1.89	0.50	−3.75^b^	0.15 (84.90)
	Capability	0.03	0.08	0.37	1.03 (3.20)
	Opportunity	0.03	0.08	0.41	1.03 (3.20)
	Motivation	0.15	0.07	2.05^d^	1.17 (16.60)
	Flare-ups	0.96	0.32	3.01^c^	2.62 (100)
Graded tasks	(Intercept)	−4.73	0.75	−6.29^b^	0.01 (99.10)
	Capability	0.06	0.09	0.59	1.06 (5.70)
	Opportunity	0.20	0.09	2.32^d^	1.22 (22.40)
	Motivation	0.45	0.09	5.05^b^	1.57 (57.20)
	Commitment	0.11	0.31	0.35	1.12 (11.70)
Action planning	(Intercept)	−3.98	0.73	−5.42^b^	0.02 (98.10)
	Capability	0.11	0.10	1.13	1.12 (11.80)
	Opportunity	0.21	0.09	2.29^d^	1.23 (23.40)
	Motivation	0.18	0.09	2.10^d^	1.20 (19.60)
	Ethnicity	−0.81	0.55	−1.47	0.45 (55.40)
Problem solving	(Intercept)	−4.25	0.80	−5.28^b^	0.01 (98.60)
	Capability	0.21	0.10	2.13^d^	1.23 (23.00)
	Opportunity	0.06	0.09	0.73	1.07 (6.50)
	Motivation	0.16	0.08	1.97^d^	1.18 (17.90)
	Flare-ups	−0.66	0.38	−1.72	0.52 (48.30)
	Gender	1.29	0.58	2.24^d^	3.63 (100)
Observe demonstration of physical activity	Intercept	−3.95	0.73	−5.40^b^	0.02 (98.10)
	Capability	−0.03	0.10	−0.25	0.98 (2.50)
	Opportunity	0.25	0.10	2.60^c^	1.28 (28.30)
	Motivation	0.27	0.09	3.14^c^	1.32 (31.50)
	Flare-ups	−0.39	0.37	−1.05	0.67 (32.60)
	Commitment	−0.30	0.31	−0.98	0.74 (26.20)
Self-talk	(Intercept)	−4.51	0.97	−4.67^b^	0.02 (97.90)
	Capability	−0.14	0.11	−1.33	0.91 (8.80)
	Opportunity	0.24	0.10	2.35^d^	1.21 (21)
	Motivation	0.47	0.10	4.53^b^	1.62 (62.20)
	Gender	1.08	0.71	1.53	1.08 (8.30)
	Commitment	−0.35	0.34	−1.02	0.70 (30.40)
Finding meaning in physical activity	(Intercept)	−2.26	0.61	−3.72^b^	0.11 (89.50)
	Capability	0.00	0.09	0.04	1.00 (0.40)
	Opportunity	0.12	0.09	1.44	1.13 (13)
	Motivation	0.36	0.09	4.24^b^	1.44 (43.90)
	Illness	−1.13	0.46	−2.44^d^	0.32 (67.70)
	Commitment	−0.19	0.32	−0.61	0.82 (17.60)
Focus on past success	Intercept	−4.36	0.79	−5.52^b^	0.03 (97.10)
	Capability	0.04	0.10	0.38	1.16 (15.70)
	Opportunity	0.11	0.09	1.22	1.35 (35.40)
	Motivation	0.44	0.10	4.57^b^	1.30 (29.50)
	Commitment	−0.23	0.33	−0.70	0.95 (4.80)
Self-reward (behavior)	(Intercept)	−5.85	0.97	−6.05^b^	0.00 (99.70)
	Capability	0.16	0.10	1.58	1.18 (17.80)
	Opportunity	0.13	0.09	1.43	1.14 (14.10)
	Motivation	0.33	0.10	3.37^b^	1.39 (38.70)
	Gender	1.51	0.70	2.17^d^	4.52 (100)
Emotional support	(Intercept)	4.38	0.79	−5.57^b^	0.01 (98.74)
	Capability	−0.01	0.10	−0.12	0.99 (1.25)
	Opportunity	0.44	0.10	4.40^b^	1.55 (54.65)
	Motivation	0.12	0.09	1.33	1.13 (12.92)
	Ethnicity	0.11	0.61	0.18	1.12 (11.54)

a % Change is calculated from the odds ratio. Odds > 1 = (odds ratio − 1*100). Odds < 1 = (1 − odds ratio*100).

b *P* < .001.

c *P* < .01.

d *P* < .05.

## Discussion

Our study aimed to describe the enactment of BCTs and investigate their key predictors among physically active university students living with physical disabilities and chronic conditions over 10 days. By examining daily BCT enactment, our study identified which BCTs were used more frequently and the demographic (eg, gender) and contextual variables (eg, flare-up) that predicted enactment of specific BCTs. Further, motivation and opportunity were found to be a more common predictor of BCT enactment compared to capability. Given the general lack of knowledge of BCT enactment, especially among individuals with physical disabilities/chronic conditions, these findings provide valuable insights to the behavior change literature. Specifically, our findings provide key considerations for selecting and integrating BCTs in physical activity interventions for this population.

Participants in our study reported an average of 245.6 minutes/week of LTPA and 208.2 minutes/week of MVPA, based on daily average 49.11 and 41.64 minutes, over an average of 5 active days within a 10-day study period. Participants achieving or exceeding the physical activity guidelines likely reflect our recruitment of students with disabilities who were already physically active. The values of our study among university students with physical disability and chronic conditions are generally comparable to those reported by Úbeda-Colomer et al^36^ and Pans et al,^37^ where MVPA ranged from 177 to 279 minutes/week. A few contextual differences may explain the slighter bigger physical activity levels from our study. Our participants were already physically active and living in a city known for its walkability. While not dramatically higher, the aforementioned factors may have contributed to higher physical activity levels compared to the broader Spanish university populations.

The descriptive analysis on daily enactment of BCTs demonstrated that participants used an average of 11 BCTs each day and enacted 22 distinct BCTs over 10 days. Hankonen et al,^11^ who retrospectively assessed BCT enactment 1 year postintervention among adults with type 2 diabetes, found that participants who used 6-7 BCTs increased their physical activity levels. The authors claimed that while using more BCTs might lead to higher physical activity levels compared to groups who use less, simply increasing the number of BCTs does not guarantee behavior change outcomes. However, our findings suggest that “more is likely happening” as physically active students with physical disabilities and chronic conditions report enacting more than 7 BCTs. Therefore, more research is needed before fully concluding the ideal number of BCTs, and how to facilitate the daily integration of multiple BCTs to promote behavior change outside of controlled environments.

Consistent with Hankonen et al,^12^ motivational BCTs (eg, reflecting on motives and values) were reported more frequently than self-regulatory BCTs (eg, self-monitoring). This may reflect the greater cognitive demands of self-regulatory BCTs,^13^^,^^14^^,^^38^^,^^39^ which may not align with the readiness or needs of already active individuals. As noted by Wee and Dillon,^38^ the effectiveness of self-regulatory strategies may vary based on an individual’s stage of change, and in some cases, may introduce unnecessary cognitive burden. Further research is needed to examine differences in enactment, cognitive load, and relevance of motivational versus self-regulatory BCTs across behavioral stages.

In the behavior change literature, it is unclear how different demographic groups use BCTs. Our study explores these nuances, specifically looking at gender and disability differences in BCT enactment. Our findings show that women are more likely than men to enact 13 different self-regulatory BCTs (eg, self-monitoring and problem solving). Our findings align with another study, reporting that women are more incline in enacting self-regulatory strategies than men.^40^ While this difference could stem from numerous factors (eg, emotional intelligence),^41^ further investigation into gender differences in BCT enactment is necessary before drawing any final conclusions. Additionally, our study found that the types of BCTs used by students with physical disabilities and those with chronic conditions were generally similar, with differences on only 2 of the BCTs. It appears logical to research these 2 groups together when examining BCT enactment for physical activity participation. Although the population does not differ in BCT usage, students with physical disabilities and chronic conditions have reported that when commitments (eg, health appointments) and flareups (eg, health-related symptoms) are present, the likelihood of using some BCTs decreases. These results are expected given the contextual barriers individuals with disabilities often face and their impact on health behaviors.^4^^,^^27^^,^^28^ Thus, since both gender differences and contextual factors have an impact on BCT enactment they should be carefully considered in the examination and application of BCTs.

While examining student’s capability, opportunity, and motivation in using BCTs, we found that students reported more capability and opportunity to use BCTs daily than motivation. Despite this, motivation was found to be the strongest predictor of the enactment of most BCTs. Similar to our findings, Palsola et al^13^ qualitatively explored BCT enactment and found that, despite participants being knowledgeable about BCTs (ie, high psychological capability), they often found self-regulatory BCTs (eg, self-monitoring and action planning) demotivating as some were tedious, time-consuming (ie, low physical opportunity), and felt like chores rather than enjoyable skills (ie, low automatic motivation). Additionally, it could clarify why motivational BCTs (eg, thinking about one’s motives and values) were more frequently reported instead, as they may be perceived as less burdensome, easier to enact, and inherently motivational, a finding also noted by Hankonen et al^12^ Therefore, our findings support the notion that BCTs have their own psychological predictors, as proposed by Hankonen.^11^ However, as highlighted in other studies,^12–14^^,^^38^^,^^39^ the psychological process of BCT enactment is complex, and further in-depth examination is needed to fully capture the nuances in BCT enactment. Such theoretical understanding can guide the development of future behavior change interventions, potentially facilitating the enactment of BCTs and rendering interventions more effective.

## Conclusion

Our study samples were mostly female and white, though it aligns with the broader Canadian University demographic of 66% female, and 42% self-identified as a racialized person.^5^ Further, our study and future studies would benefit from differentiating between individuals with congenital disabilities/chronic conditions (ie, disabilities present from birth), and those with acquired disabilities/chronic conditions (ie, occurring later in life) as they differ in physical activity engagement.^4^ Some limitations in our study include reporting of implicit BCT use instead of explicit BCT enactment due to questionnaire design. Further, while our BCT selection does not capture the full spectrum of BCTs, it provides an excellent first step in advancing our understanding of BCT enactment. Additionally, BCT enactment was measured dichotomously (ie, BCT usage; Yes/No), and employing qualitative methods, such as those used by French et al,^14^ could provide greater depth of the participant’s experience of enacting BCT. Further, our study focused on describing BCT enactment as the primary outcome, but future research should examine the potential mediational pathways between theoretical variables, BCT enactment, and physical activity. We acknowledge that conducting multiple analyses increases the chance to find statistically significant results; nonetheless, our study provides a valuable preliminary step to understand BCT enactment.

##  

## Supplementary Material

kaaf065_Supplementary_Data

## Data Availability

Deidentified data from this study are not available in a public archive. Deidentified data from this study will be made available (as allowable according to institutional IRB standards) by emailing the corresponding author.

## Transparency statements

(1) This study was not formally pre-registered. (2) The analysis plan was not formally pre-registered. (3) Analytic code used to conduct the analyses presented in this study are available in a public archive: https://cran.r-project.org/web/packages/lme4/lme4.pdf. (4) Some of the materials used to conduct the study are presented in a public archive: Leisure-time Physical Activity: https://fhsd-sciactioncanada-2019.sites.olt.ubc.ca/files/2024/06/LTPAQ-D-Protocol_2024.pdf. For more information on the development, validity and reliability of the LTPAQ-D, please see the published paper freely available at https://www.mdpi.com/2673-7272/4/2/21. Gee CM, Neely A, Jevdjevic A, Olsen K, Martin Ginis KA. The leisure time physical activity questionnaire for people with disabilities: validity and reliability. *Disabilities* 2024;4:321-331.
