# Supplementary material for: The daily enactment of behavior change techniques among physically active university students with physical disabilities and chronic conditions
Source: Ann Behav Med. 2025 Aug 26;59(1):kaaf065. doi: 10.1093/abm/kaaf065 (PMC12380471; doi:10.1093/abm/kaaf065)
Supplement: kaaf065_Supplementary_Data [file kaaf065_supplementary_data.docx]

# **Supplementary Materials**

**Supplementary Material Table 1.**

Odds Ratios and Percentage Changes for Socio-Demographic Predictors in BCTs from GLMM

| Model | Predictors | | Estimate (Log-Odds) | Std. Error | Z value | Odd ratios |
| --- | --- | --- | --- | --- | --- | --- |
| Self-Monitoring | | (Intercept) | -2.39 | 0.75 | -3.19** | 0.09 |
|  | | Gender | 2.76 | 1.00 | 2.77** | 15.84 |
|  | | Ethnicity | -2.22 | 0.88 | 0.18* | 0.11 |
|  | | Disability | 0.14 | 0.80 | 0.86 | 1.15 |
| Obtaining Information about Health Consequences | | (Intercept) | -2.52 | 0.56 | -4.48*** | 0.08 |
|  | | Gender | 2.40 | 0.74 | 3.24** | 11.00 |
|  | | Ethnicity | -1.91 | 0.65 | -2.95** | 0.15 |
|  | | Disability | 0.27 | 0.58 | 0.46 | 1.31 |
| Antecedents | | (Intercept) | -1.77 | 0.54 | -3.28** | 0.17 |
|  | | Gender | 1.48 | 0.75 | 1.98* | 4.38 |
|  | | Ethnicity | -1.16 | 0.68 | -1.72 | 0.31 |
|  | | Disability | -0.34 | 0.62 | -0.56 | 0.71 |
| Task Crafting | | (Intercept) | 0.76 | 0.36 | 2.11* | 2.15 |
|  | | Gender | 0.36 | 0.52 | 0.69 | 1.43 |
|  | | Ethnicity | -0.15 | 0.48 | -0.32 | 0.86 |
|  | | Disability | -0.06 | 0.45 | -0.14 | 0.94 |
| Practical Social Support | | (Intercept) | -2.51 | 0.54 | -4.61*** | 0.08 |
|  | | Gender | 1.42 | 0.72 | 1.97* | 4.16 |
|  | | Ethnicity | -0.01 | 0.63 | -0.01 | 0.99 |
|  | | Disability | -0.36 | 0.59 | -0.61 | 0.70 |
| Obtaining Information on How to Perform Physical Activity | | (Intercept) | -1.86 | 0.58 | -3.18** | 0.16 |
|  | | Gender | 0.26 | 0.81 | 0.32 | 1.30 |
|  | | Ethnicity | -0.27 | 0.72 | -0.38 | 0.76 |
|  | | Disability | 0.36 | 0.65 | 0.56 | 1.44 |
| Restructuring the Social Environment | | (Intercept) | -0.53 | 0.38 | -1.39 | 0.59 |
|  | | Gender | 1.38 | 0.55 | 2.51* | 3.97 |
|  | | Ethnicity | -0.38 | 0.50 | -0.76 | 0.69 |
|  | | Disability | -1.05 | 0.47 | -2.22* | 0.35 |
| Restructuring the Physical Environment | | (Intercept) | -1.05 | 0.47 | -2.21* | 0.35 |
|  | | Gender | 2.01 | 0.67 | 3.01** | 7.46 |
|  | | Ethnicity | -0.82 | 0.60 | -1.37 | 0.44 |
|  | | Disability | -0.64 | 0.56 | -1.15 | 0.52 |
| Prompt and Cues | | (Intercept) | 1.85 | 0.43 | -4.36*** | 0.16 |
|  | | Gender | 1.84 | 0.57 | 3.23** | 6.31 |
|  | | Ethnicity | 0.16 | 0.49 | 0.33 | 1.18 |
|  | | Disability | -1.20 | 0.47 | -2.53* | 0.30 |
| Behavioural Goals | | (Intercept) | -0.17 | 0.38 | -0.43 | 0.85 |
|  | | Gender | 0.25 | 0.55 | 0.46 | 1.29 |
|  | | Ethnicity | 0.28 | 0.50 | -0.55 | 0.76 |
|  | | Disability | 0.48 | 0.46 | 1.03 | 1.61 |
| Outcome Goals | | (Intercept) | -0.46 | 0.46 | -1.01 | 0.63 |
|  | | Gender | 0.04 | 0.66 | 0.06 | 1.04 |
|  | | Ethnicity | -0.63 | 0.60 | -1.05 | 0.53 |
|  | | Disability | 0.80 | 0.55 | 1.45 | 2.23 |
| Goal Integration | | (Intercept) | 0.57 | 0.45 | 1.27 | 1.77 |
|  | | Gender | 0.60 | 0.65 | 0.93 | 1.83 |
|  | | Ethnicity | -0.30 | 0.59 | -0.52 | 0.74 |
|  | | Disability | -0.21 | 0.55 | -0.37 | 0.81 |
| Positive Reframing | | (Intercept) | 0.26 | 0.52 | -0.50 | 0.77 |
|  | | Gender | 1.54 | 0.75 | 2.06* | 4.67 |
|  | | Ethnicity | -0.45 | 0.68 | -0.66 | 0.64 |
|  | | Disability | -0.52 | 0.63 | -0.84 | 0.59 |
| Pros and Cons | | (Intercept) | -3.10 | 0.74 | -4.18 | 0.05 |
|  | | Gender | 1.54 | 0.97 | 1.59 | 4.67 |
|  | | Ethnicity | -1.22 | 0.88 | -1.39 | 0.30 |
|  | | Disability | 0.00 | 0.82 | 0.00 | 1.00 |
| Reflection on the Need to Perform Physical Activity | | (Intercept) | -0.69 | 0.52 | -1.32 | 0.50 |
|  | | Gender | 0.67 | 0.75 | 0.89 | 1.96 |
|  | | Ethnicity | -1.01 | 0.70 | -1.45 | 0.36 |
|  | | Disability | 0.34 | 0.65 | 0.52 | 1.40 |
| Finding Meaning in Physical Activity | | (Intercept) | 0.09 | 0.52 | 0.17 | 1.09 |
|  | | Gender | 0.86 | 0.75 | 1.14 | 2.35 |
|  | | Ethnicity | -0.84 | 0.69 | -1.22 | 0.43 |
|  | | Disability | 0.32 | 0.63 | 0.50 | 1.37 |
| Self-Talk | | (Intercept) | -1.03 | 0.54 | -1.91 | 0.36 |
|  | | Gender | 2.10 | 0.79 | 2.65** | 8.18 |
|  | | Ethnicity | -1.06 | 0.70 | -1.51 | 0.35 |
|  | | Disability | -1.12 | 0.65 | -1.73 | 0.33 |
| Behavioural Self-Praise | | (Intercept) | -1.56 | 0.60 | -2.58** | 0.21 |
|  | | Gender | 2.03 | 0.85 | 2.40* | 7.65 |
|  | | Ethnicity | -1.76 | 0.77 | -2.27* | 0.17 |
|  | | Disability | -0.59 | 0.71 | -0.83 | 0.56 |
| Behavioural Self-Reward | | (Intercept) | -1.63 | 0.52 | -3.14** | 0.20 |
|  | | Gender | 1.75 | 0.72 | 2.43* | 5.76 |
|  | | Ethnicity | -0.63 | 0.63 | -0.99 | 0.53 |
|  | | Disability | -0.33 | 0.58 | -0.57 | 0.72 |
| Normalizing Difficulty | | (Intercept) | -0.09 | 0.43 | -0.21 | 0.91 |
|  | | Gender | 0.51 | 0.61 | 0.84 | 1.67 |
|  | | Ethnicity | -0.89 | 0.57 | -1.57 | 0.41 |
|  | | Disability | 0.09 | 0.52 | 0.18 | 1.10 |
| Focus on Enjoyment | | (Intercept) | -0.01 | 0.37 | -0.03 | 0.99 |
|  | | Gender | 1.05 | 0.54 | 1.94 | 2.85 |
|  | | Ethnicity | 0.18 | 0.50 | 0.37 | 1.20 |
|  | | Disability | -0.47 | 0.47 | -1.00 | 0.63 |
| Self-Kindness | | (Intercept) | -0.99 | 0.40 | -2.51* | 0.37 |
|  | | Gender | 0.84 | 0.55 | 1.53 | 2.32 |
|  | | Ethnicity | -0.27 | 0.50 | -0.54 | 0.76 |
|  | | Disability | 0.31 | 0.46 | 0.67 | 1.36 |
| Graded Task | | (Intercept) | -0.99 | 0.40 | -2.51* | 0.37 |
|  | | Gender | 0.84 | 0.55 | 1.53 | 2.32 |
|  | | Ethnicity | -0.27 | 0.50 | -0.54 | 0.76 |
|  | | Disability | 0.31 | 0.46 | 0.67 | 1.36 |
| Action Planning | | (Intercept) | -1.50 | 0.50 | 0.56** | 0.22 |
|  | | Gender | 1.24 | 0.69 | 1.81 | 3.45 |
|  | | Ethnicity | -1.37 | 0.62 | -2.22* | 0.25 |
|  | | Disability | 0.49 | 0.56 | 0.87 | 1.63 |
| Emotional Support | | (Intercept) | -1.67 | 0.54 | -3.09** | 0.19 |
|  | | Gender | 1.73 | 0.75 | 2.30* | 5.65 |
|  | | Ethnicity | -0.57 | 0.67 | -0.85 | 0.56 |
|  | | Disability | -0.24 | 0.63 | -0.37 | 0.79 |
| Problem Solving | | (Intercept) | -1.43 | 0.47 | -3.05** | 0.24 |
|  | | Gender | 1.60 | 0.65 | 2.45* | 4.93 |
|  | | Ethnicity | -0.51 | 0.58 | -0.88 | 0.60 |
|  | | Disability | -0.43 | 0.54 | -0.81 | 0.65 |
| Focus on Past Success | | (Intercept) | -1.13 | 0.51 | -2.20* | 0.32 |
|  | | Gender | 1.10 | 0.72 | 1.53 | 3.02 |
|  | | Ethnicity | -1.12 | 0.66 | -1.70 | 0.33 |
|  | | Disability | 0.25 | 0.60 | 0.42 | 1.29 |
| Observe Demonstration of Physical Activity | | (Intercept) | -1.21 | 0.43 | -2.85** | 0.30 |
|  | | Gender | 0.97 | 0.60 | 1.61 | 2.63 |
|  | | Ethnicity | -0.35 | 0.55 | -0.64 | 0.70 |
|  | | Disability | -0.77 | 0.51 | -1.50 | 0.46 |

*_a. %_* _Change is calculated from the odds ratio. Odds > 1 = (odds ratio - 1*100). Odds < 1 = (1- odds ratio *100). * = p <0.05, ** = p < 0.01, *** = p < 0.001_

**Supplementary Material Table 2.**

Odds Ratios and Percentage Changes for Contextual Factors Predictors in BCTs Techniques from GLMM

| Model | Predictors | Estimate (Log-Odds) | Std. Error | Z value | Odd ratios |
| --- | --- | --- | --- | --- | --- |
| Self-Monitoring | (Intercept) | -1.00 | 0.43 | -2.31* | 0.37 |
|  | Weather | -0.26 | 0.46 | -0.57 | 0.77 |
|  | Illness | -0.07 | 0.51 | -0.14 | 0.93 |
|  | Flareup | -0.82 | 0.43 | -1.89 | 0.44 |
|  | Commitments | -1.00 | 0.36 | -2.76** | 0.37 |
|  | Exams | 0.09 | 0.41 | 0.22 | 1.09 |
| Obtaining Information about Health Consequences | (Intercept) | -1.57 | 0.33 | -4.71* | 0.79 |
|  | Weather | 0.41 | 0.41 | 1.00 | 0.50 |
|  | Illness | -0.03 | 0.49 | -0.06 | 0.03 |
|  | Flareup | 0.60 | 0.39 | -1.53 | 0.45 |
|  | Commitments | 0.01 | 0.32 | 0.05 | 0.01 |
|  | Exams | -0.41 | 0.42 | -0.99 | 0.34 |
| Antecedents | (Intercept) | -1.29 | 0.34 | -3.84*** | 0.28 |
|  | Weather | -0.21 | 0.42 | -0.49 | 0.81 |
|  | Illness | -0.25 | 0.49 | -0.52 | 0.78 |
|  | Flareup | -0.40 | 0.38 | -1.04 | 0.67 |
|  | Commitments | -0.28 | 0.32 | -0.89 | 0.75 |
|  | Exams | -0.39 | 0.41 | -0.95 | 0.68 |
| Practical Support | (Intercept) | -1.57 | 0.33 | -4.79*** | -2.57 |
|  | Weather | -0.28 | 0.42 | -0.67 | 1.28 |
|  | Illness | 0.12 | 0.39 | 0.32 | 0.92 |
|  | Flareup | -0.13 | 0.37 | -0.36 | 0.13 |
|  | Commitments | -0.13 | 0.32 | -0.42 | 1.13 |
|  | Exams | -0.49 | 0.42 | -1.15 | 1.49 |
| Task Crafting | (Intercept) | 1.69 | 0.27 | 6.26*** | 5.42 |
|  | Weather | -0.33 | 0.37 | -0.90 | 0.72 |
|  | Illness | 0.12 | 0.39 | 0.32 | 1.13 |
|  | Flareup | -1.07 | 0.32 | -3.35*** | 0.34 |
|  | Commitments | -1.07 | 0.28 | -3.78*** | 0.34 |
|  | Exams | -0.31 | 0.33 | -0.95 | 0.73 |
| Obtaining Information on How to Perform Physical Activity | (Intercept) | -1.07 | 0.35 | -3.11** | 0.34 |
|  | Weather | -0.92 | 0.50 | -1.84 | 0.40 |
|  | Illness | -0.94 | 0.59 | -1.61 | 0.39 |
|  | Flareup | -0.78 | 0.43 | -1.80 | 0.46 |
|  | Commitments | -1.03 | 0.37 | -2.80** | 0.36 |
|  | Exams | -0.06 | 0.44 | -0.12 | 0.95 |
| Restructuring the Social Environment | (Intercept) | 0.13 | 0.25 | 0.53 | 1.14 |
|  | Weather | -0.09 | 0.35 | -0.25 | 0.92 |
|  | Illness | -0.02 | 0.38 | -0.05 | 0.98 |
|  | Flareup | -0.67 | 0.31 | -2.18* | 0.51 |
|  | Commitments | -0.38 | 0.27 | -1.44 | 0.68 |
|  | Exams | -0.23 | 0.33 | -0.72 | 0.79 |
| Restructuring the Physical Environment | (Intercept) | 0.13 | 0.31 | 0.43 | 1.14 |
|  | Weather | 0.29 | 0.38 | 0.76 | 1.34 |
|  | Illness | -0.15 | 0.42 | -0.37 | 0.86 |
|  | Flareup | -0.54 | 0.34 | -1.60 | 0.58 |
|  | Commitments | -0.87 | 0.29 | -3.02** | 0.42 |
|  | Exams | -0.53 | 0.35 | -1.50 | 0.59 |
| Prompt and Cues | (Intercept) | -0.28 | 0.29 | -0.98 | 0.75 |
|  | Weather | -0.75 | 0.43 | -1.74 | 0.47 |
|  | Illness | -0.78 | 0.47 | -1.65 | 0.46 |
|  | Flareup | -1.03 | 0.37 | -2.78** | 0.36 |
|  | Commitments | -1.01 | 0.32 | -3.20** | 0.36 |
|  | Exams | 0.19 | 0.36 | 0.53 | 1.21 |
| Behavioural Goals | (Intercept) | 0.65 | 0.24 | 2.65** | 1.91 |
|  | Weather | -0.33 | 0.36 | -0.92 | 0.72 |
|  | Illness | 0.05 | 0.39 | 0.12 | 1.05 |
|  | Flareup | -0.54 | 0.30 | -1.77 | 0.58 |
|  | Commitments | -1.05 | 0.27 | 3.85*** | 0.35 |
|  | Exams | -0.38 | 0.33 | -1.17 | 0.68 |
| Outcome Goals | (Intercept) | -0.03 | 0.27 | -0.13 | 0.97 |
|  | Weather | -0.07 | 0.37 | -0.18 | 0.94 |
|  | Illness | -0.75 | 0.42 | -1.80 | 0.47 |
|  | Flareup | -0.41 | 0.32 | -1.26 | 0.66 |
|  | Commitments | -0.47 | 0.28 | -1.71 | 0.62 |
|  | Exams | -0.38 | 0.35 | -1.11 | 0.68 |
| Goal Integration | (Intercept) | 1.16 | 0.27 | 4.28*** | 3.20 |
|  | Weather | 0.08 | 0.37 | 0.22 | 1.08 |
|  | Illness | -0.42 | 0.39 | -1.07 | 0.66 |
|  | Flareup | -0.45 | 0.31 | -1.44 | 0.64 |
|  | Commitments | -0.58 | 0.27 | -2.11* | 0.56 |
|  | Exams | -0.43 | 0.33 | -1.29 | 0.65 |
| Positive Reframing | (Intercept) | 0.74 | 0.33 | 2.29* | 2.10 |
|  | Weather | -0.70 | 0.39 | -1.80 | 0.50 |
|  | Illness | 0.55 | 0.42 | 1.31 | 1.73 |
|  | Flareup | -0.69 | 0.34 | -2.05* | 0.50 |
|  | Commitments | -0.25 | 0.29 | -0.86 | 0.78 |
|  | Exams | -0.22 | 0.36 | -0.61 | 0.80 |
| Pros and Cons | (Intercept) | -2.81 | 0.47 | -5.99*** | 0.06 |
|  | Weather | 0.26 | 0.45 | 0.57 | 1.30 |
|  | Illness | -0.82 | 0.59 | -1.40 | 0.44 |
|  | Flareup | 0.64 | 0.41 | 1.56 | 1.89 |
|  | Commitments | 0.34 | 0.36 | 0.95 | 1.40 |
|  | Exams | -0.49 | 0.48 | -1.02 | 0.61 |
| Reflection on the Need to Perform Physical Activity | (Intercept) | -0.34 | 0.30 | -1.11 | 0.71 |
|  | Weather | -0.06 | 0.39 | -0.17 | 0.94 |
|  | Illness | 0.16 | 0.42 | 0.38 | 1.17 |
|  | Flareup | -0.24 | 0.34 | -0.72 | 0.78 |
|  | Commitments | -0.73 | 0.29 | -2.49* | 0.48 |
|  | Exams | 0.28 | 0.36 | 0.77 | 1.32 |
| Behavioural Self-Praise | (Intercept) | -0.84 | 0.34 | -2.45* | 0.43 |
|  | Weather | 0.10 | 0.40 | 0.26 | 1.11 |
|  | Illness | -0.06 | 0.44 | -0.12 | 0.95 |
|  | Flareup | -0.53 | 0.37 | -1.45 | 0.59 |
|  | Commitments | -0.73 | 0.31 | -2.33* | 0.48 |
|  | Exams | -0.15 | 0.40 | -0.37 | 0.86 |
| Normalizing Difficulty | (Intercept) | 0.06 | 0.26 | 0.22 | 1.06 |
|  | Weather | -0.02 | 0.35 | -0.06 | 0.98 |
|  | Illness | 0.11 | 0.39 | 0.29 | 1.12 |
|  | Flareup | -0.30 | 0.31 | -0.98 | 0.74 |
|  | Commitments | -0.60 | 0.27 | -2.22* | 0.55 |
|  | Exams | 0.37 | 0.34 | 1.11 | 1.45 |
| Focus on Enjoyment | (Intercept) | 1.27 | 0.29 | 4.41*** | 3.55 |
|  | Weather | -0.27 | 0.37 | -0.73 | 0.76 |
|  | Illness | -0.58 | 0.40 | -1.45 | 0.56 |
|  | Flareup | -0.78 | 0.32 | -2.42* | 0.46 |
|  | Commitments | -0.63 | 0.28 | -2.22* | 0.53 |
|  | Exams | -0.67 | 0.35 | -1.94 | 0.51 |
| Self-Kindness | (Intercept) | -0.42 | 0.25 | -1.67 | 0.66 |
|  | Weather | -0.08 | 0.35 | -0.24 | 0.92 |
|  | Illness | -0.53 | 0.38 | -1.37 | 0.59 |
|  | Flareup | 0.72 | 0.30 | 2.37* | 2.05 |
|  | Commitments | 0.01 | 0.27 | 0.03 | 1.01 |
|  | Exams | -0.49 | 0.35 | -1.41 | 0.61 |
| Graded Task | (Intercept) | 0.00 | 0.25 | 0.02 | 1.00 |
|  | Weather | -0.05 | 0.35 | -0.14 | 0.95 |
|  | Illness | -0.44 | 0.39 | -1.12 | 0.64 |
|  | Flareup | -0.30 | 0.31 | -0.98 | 0.74 |
|  | Commitments | -0.56 | 0.27 | -2.07* | 0.57 |
|  | Exams | -0.28 | 0.33 | -0.84 | 0.76 |
| Action Planning | (Intercept) | -0.95 | 0.31 | -3.06** | 1.47 |
|  | Weather | -0.36 | 0.44 | -0.83 | 2.00 |
|  | Illness | -0.94 | 0.50 | -1.88 | 1.48 |
|  | Flareup | 0.01 | 0.36 | 0.03 | 2.74 |
|  | Commitments | -0.50 | 0.31 | -1.62 | 1.84 |
|  | Exams | 0.04 | 0.38 | 0.09 | 2.82 |
| Emotional Support | (Intercept) | -0.51 | 0.31 | -1.64 | 0.40 |
|  | Weather | -0.11 | 0.37 | -0.30 | 0.10 |
|  | Illness | 0.05 | 0.41 | 0.12 | 0.05 |
|  | Flareup | -0.29 | 0.33 | -0.90 | 0.25 |
|  | Commitments | -0.38 | 0.29 | -1.33 | 0.32 |
|  | Exams | -0.57 | 0.36 | -1.58 | 0.43 |
| Problem Solving | (Intercept) | -0.34 | 0.27 | -1.23 | 0.71 |
|  | Weather | -0.18 | 0.37 | -0.48 | 0.84 |
|  | Illness | -0.10 | 0.42 | -0.23 | 0.91 |
|  | Flareup | -0.99 | 0.34 | -2.94** | 0.37 |
|  | Commitments | -0.26 | 0.28 | -0.91 | 0.77 |
|  | Exams | -0.36 | 0.36 | -1.02 | 0.70 |
| Observe Demonstration of Physical Activity | (Intercept) | -0.55 | 0.26 | -2.10* | 0.57 |
|  | Weather | 0.00 | 0.38 | 0.00 | 1.00 |
|  | Illness | 0.00 | 0.43 | -0.01 | 1.00 |
|  | Flareup | -0.91 | 0.35 | -2.58** | 0.40 |
|  | Commitments | -0.86 | 0.30 | -2.88** | 0.42 |
|  | Exams | -0.07 | 0.37 | -0.20 | 0.93 |
| Self -Talk | (Intercept) | -0.12 | 0.32 | -0.36 | 0.89 |
|  | Weather | 0.27 | 0.38 | 0.71 | 1.31 |
|  | Illness | 0.03 | 0.42 | 0.06 | 1.03 |
|  | Flareup | -0.54 | 0.34 | -1.62 | 0.58 |
|  | Commitments | -1.01 | 0.30 | -3.37*** | 0.37 |
|  | Exams | 0.07 | 0.36 | 0.20 | 1.07 |
| Finding Meaning in Physical Activity | (Intercept) | 0.88 | 0.32 | 2.80** | 2.42 |
|  | Weather | -0.54 | 0.38 | -1.43 | 0.58 |
|  | Illness | -1.34 | 0.44 | -3.02** | 0.26 |
|  | Flareup | -0.05 | 0.34 | -0.15 | 0.95 |
|  | Commitments | -0.67 | 0.30 | -2.24* | 0.51 |
|  | Exams | -0.16 | 0.37 | -0.42 | 0.85 |
| Focus on Past Success | (Intercept) | -0.49 | 0.29 | -1.71 | -0.49 |
|  | Weather | 0.14 | 0.38 | 0.36 | 0.14 |
|  | Illness | -0.34 | 0.43 | -0.78 | -0.34 |
|  | Flareup | -0.11 | 0.34 | -0.33 | -0.11 |
|  | Commitments | -0.78 | 0.29 | -2.70** | -0.78 |
|  | Exams | -0.47 | 0.38 | -1.23 | -0.47 |
| Behavioural Self-Reward | (Intercept) | -0.27 | 0.29 | -0.94 | 0.76 |
|  | Weather | -0.31 | 0.39 | -0.81 | 0.73 |
|  | Illness | -0.17 | 0.43 | -0.39 | 0.85 |
|  | Flareup | -0.78 | 0.34 | -2.28 | 0.46 |
|  | Commitments | -0.88 | 0.29 | -3.01 | 0.41 |
|  | Exams | 0.08 | 0.36 | 0.23 | 1.08 |

*^a. %^* ^Change is calculated from the odds ratio. Odds > 1 = (odds ratio - 1*100). Odds < 1 = (1- odds ratio *100). * = p <0.05, ** = p < 0.01, *** = p < 0.001^

**Supplementary Material Table 3.**

*BCT Selection Process*

| Step | Description | BCTs |
| --- | --- | --- |
| Step One | Conducted a literature search in PsycINFO, PubMed, and Medline to identify BCTs in physical activity interventions from 26 meta-analyses/reviews. Selected 8 BCTs based on frequency and effectiveness. | **Chosen BCTs:**   - Social support - Self-monitoring - Goal setting - Action planning - Problem-solving - Graded task - Credible source - Goal setting behavior |
| Step Two | Consulted the self-enactable compendium list. Identified 13 new chosen BCTs | **Chosen BCTs:**   - Behavioural goals - Outcome goals - Action planning - Problem-solving - Self-monitoring of behavior - Restructuring physical environment - Goal integration - Focus on the enjoyment of the behavior - Obtain emotional support - Prompt/cues - Task Crafting - Normalize difficulty - Graded Tasks |
| Step Tree | Aligned the 13 BCTs with the theoretical domain framework and COM-B model. Identified 15 new BCTs to cover all domains. | **Chosen BCTs:**   - Self-kindness - Self-praise (behavior) - Self-reward (behavior) - Observing the demonstration of the behavior - Self-talk - Focusing on past success - Finding meaning in the target behavior - Reflecting on the need to perform the behavior - Pros and cons - Positive reframing - Restructuring the social environment - Obtain instruction on how to perform the behavior - Obtain practical social support - Obtain information about antecedents - Obtain information about health consequences |
| Total of BCTs (28) |  | - Behavioural goals - Outcome goals - Action planning - Problem-solving - Self-monitoring of behavior - Restructuring physical environment - Goal integration - Focus on the enjoyment of the behavior - Obtain emotional support - Prompt/cues - Task Crafting - Self-praise (behavior) - Self-reward (behavior) - Observing the demonstration of the behavior - Self-talk - Focusing on past success - Finding meaning in the target behavior - Reflecting on the need to perform the behavior - Pros and cons - Positive reframing - Restructuring the social environment - Obtain instruction on how to perform the behavior - Obtain practical social support - Obtain information about antecedents - Obtain information about health consequences - Graded tasks - Normalizing difficulty - Self-Kindness |
| Step Four | Simplified BCT definitions and validated them with Ph.D., master's, and undergraduate students. Revised definitions based on feedback to ensure clarity and accuracy. | **Ph.D. and Master's Students (Trial 1):**  Missed BCTs:   - Graded task - Task crafting - Obtaining information about health consequences - Obtaining information about antecedents - Observing the demonstration of the behavior - Goal integration   **Undergraduate Student (Trial 1):**  Missed BCTs:   - Task crafting - Obtaining practical help - Observing the demonstration of the behavior - Focusing on enjoyment   **Ph.D. Student (Trial 2):**  Missed BCT:   - Goal integration |

*^a.^* ^BCTs = Behaviour Change Techniques. COM-B = Capability, Opportunity, Motivation-Behaviour.^
